# Impact of Indonesia’s national health insurance scheme on inequality in access to maternal health services: A propensity score matched analysis

**DOI:** 10.7189/jogh.10.010429

**Published:** 2020-06

**Authors:** Kanya Anindya, John Tayu Lee, Barbara McPake, Siswanto Agus Wilopo, Christopher Millett, Natalie Carvalho

**Affiliations:** 1 School of Population and Global Health, The University of Melbourne, Melbourne, Victoria, Australia; 2 Nossal Institute for Global Health, School of Population and Global Health, The University of Melbourne, Melbourne, Victoria, Australia; 3 Center for Reproductive Health and Department of Biostatistics, Epidemiology and Population Health, Faculty of Medicine, Public Health and Nursing, Universitas Gadjah Mada, Yogyakarta, Indonesia; 4 Public Health Policy Evaluation Unit, School of Public Health, Imperial College London, London, UK; 5 Centre for Health Policy & Global Burden of Disease Group, School of Population and Global Health, The University of Melbourne, Melbourne, Victoria, Australia

## Abstract

**Background:**

Reducing inequality in maternal, neonatal and infant mortality are key targets in the Sustainable Development Goals. This study is the first to evaluate the impact of Indonesia’s national health insurance scheme, *Jaminan Kesehatan Nasional* (JKN), on access to maternal health services by sociodemographic status.

**Methods:**

Using data from the 2017 Indonesia Demographic and Health Survey (IDHS) on women with live births in 2016-2017, we conducted propensity score matching (PSM) analysis to evaluate the association of JKN enrollment on the following maternal health care utilisation outcomes: (1) at least four antenatal care (ANC4+) visits; (2) ANC4+ visits and received essential components of ANC; (3) skilled birth attendance; (4) facility-based delivery; (5) post-natal care (PNC); and (6) PNC with skilled provider. Analyses were conducted at the national level and by economic subgroup and region of residence. Additionally, we investigated the potential negative impact of JKN on access to maternal health services among the uninsured population by looking at trends over time using data from the 2012 and 2017 IDHS.

**Results:**

Of the 5429 women who had recently given birth, 61% were insured by JKN in 2017. After matching treated and untreated women on key sociodemographic characteristics, enrollment in JKN was associated with a higher prevalence of receiving ANC4+ visits (7.4%, 95% confidence interval (CI) = 4.8-9.39); ANC4+ visits and received essential components of ANC (5.6%, 95% CI = 3.3-7.9); skilled birth attendance (3.0%, 95% CI = 1.5-4.5; facility-based delivery (10.2%, 95% CI = 7.5-12.7); PNC (4.0%, 95% CI = 2.2-5.7); PNC with skilled provider (4.5%, 95% CI = 2.6-6.5). Effect sizes were larger among the poor and those living in less-developed areas, such as Eastern Indonesia and Sulawesi, except for at least ANC4+ and received clinical components.

**Conclusions:**

Expansion of health insurance coverage was associated with reductions in sociodemographic inequalities in access to maternal health services in Indonesia. However, large differences in utilisation persist across regions and by economic subgroup. Accelerating progress toward universal health coverage may reduce health inequalities in other low and middle-income countries.

Despite declines in maternal, newborn, and child mortality since implementation of the Millennium Development Goals in 1990, these burdens remain disproportionately high among disadvantaged groups in low- and middle-income countries (LMICs) [1,2]. Achieving equitable access to high quality essential maternal health service has been identified as an important instrument for countries to reduce maternal and neonatal mortality and attain Sustainable Development Goals (SDGs) 3.1 and 3.2 [3]. Indonesia, a lower-middle income country, has one of the highest maternal mortality ratios (MMRs) in the South-East Asia Region [4] at 305 maternal deaths per 100 000 live births, with a substantially higher MMR of 489 in Eastern Indonesia [3,5,6]. Aside from the direct loss of life, a maternal death can result in profound negative health consequences for neonates and other children in the household, can lead to household economic deprivation, and productivity losses to society [7,8].

A high burden of maternal death is often linked to inequality in access to maternal health services [8-10]. Women from disadvantaged groups, including the poor and those living in rural and remote areas, often face increased financial barriers and limited access to high quality health services, resulting in lower coverage of essential maternal health care services [8-15]. These inequities also persist across regions. For example, health services are more concentrated in the more developed islands of Java-Bali, while lack of services and understaffing remains a problem in less developed regions of Sulawesi and Eastern Indonesia [14,16].

In 2014, the Indonesian government rolled out the world’s largest single-payer health insurance programme [12], *Jaminan Kesehatan Nasional* (JKN), or National Health Insurance, to achieve universal health coverage (UHC) by 2019. The programme integrates and replaces all previous fragmented social health insurance schemes, including *Jamkesmas, Jamkesda and Askeskin* [12,17]. Not only covering the poor and near-poor, JKN scheme is compulsory for all people in Indonesia, with a differentiated timeline until it covers the entire population. Participants are categorized into four groups: 1) subsidised participants or *Penerima Bantuan Iuran* (PBI)/Premium assistance beneficiaries, for the poor and near-poor; 2) salary earners and formal workers, for employees in the public and private sectors; 3) informal workers, for the non-poor who work in the informal sector; and 4) non-salaried workers [12]. Data from Social Security Agency for Health show the incline of enrolment rates from 48% in January 2014 to 85%, or almost 225 million people by the end of 2019 with around 60% among those classified as subsidised participants or poor and near-poor [18,19]. The programme provides comprehensive coverage of treatment for most outpatient and inpatient visits in public and enlisted private facilities. For maternal health services, JKN covers antenatal, delivery, and postnatal care, as well as referral services at secondary and tertiary hospitals [20]. Despite the introduction of JKN, the burden of out-of-pocket expenditure on health stood at 40% in 2018 [21], with the insured still incurring high OOPE.

Evidence on the impact of JKN on coverage of maternal health services and inequalities in service utilisation is lacking. Prior studies from Indonesia on maternal health and JKN were descriptive, based on older data, and focused on district-level populations [20,22-24]. No studies have used nationally-representative data to examine the impact of JKN across economic subgroups and regions [22,24]. A study using 2016 data found that JKN accelerates poor women's access to skilled birth attendance [20]. However, this study did not consider other maternal health care utilisation outcomes and did not assess the difference in impact across all economic subgroups and regions. We present the first study that uses recent population-level, nationally-representative data from 2017 to examine the impact of JKN on inequalities in maternal health services use across the continuum of care, from antenatal to intrapartum, to postpartum care. We further investigate how the impact of JKN on access to maternal health services varies across economic subgroups and regions of the country.

## METHODS

The Strengthening and Reporting of Observational Studies in Epidemiology (STROBE Statement) was used as a guidance in reporting this study [25].

### Data set and sample

We used cross-sectional data from the 2012 and 2017 Indonesia Demographic and Health Survey (IDHS), carried out by Statistics Indonesia (*Badan Pusat Statistik*) in collaboration with the National Population and Family Planning Board (*Badan Kependudukan dan Keluarga Berencana Nasional*) and the Ministry of Health [26]. DHS applies standardised instruments to measure key maternal service utilisation to ensure validity and comparability of results across countries. In Indonesia, the survey used a multi-stage stratified design to generate a representative sample from all 34 provinces. The 2017 IDHS was used for the main analysis, with the 2012 IDHS used to explore the time trend of service use between 2012 and 2017. There were 45 607 and 49 627 women aged 15-49 who completed the interview in 2012 IDHS and 2017 IDHS, respectively. The response rates were 95.9% (2012) and 97.8% (2017). A detailed description of the survey objectives and methods can be found elsewhere [26,27].

For the main analysis, our sample included women aged 15-49 years who had a recent live birth in 2016-2017. IDHS only recorded a complete information of maternal health services utilization for most recent live birth. For those with multiple births, only the last of multiple births are considered. Hence, each respondent was included only once in the analysis. To ensure differences in outcomes can be attributed to JKN, we removed 117 respondents (2% of the sample) who were only covered by other insurance including private insurance and employer-provided insurance plans. After removing respondents who had missing values in outcome and independent variables (0.6% of the sample), our sample consisted of 5717 respondents or 5429 respondents after weighting (sample flowchart in Figure S1 in the **Online Supplementary Document**). Sampling weight was applied to account for the multiple-stage design of DHS data set.

### Variables

#### Outcomes variables

We examined six indicators of maternal health service use: 1) at least 4 antenatal care (ANC 4+) visits; 2) ANC4+ visits and received clinical components of ANC; 3) skilled birth attendance (SBA); 4) facility-based delivery; 5) post-natal care (PNC); and 6) and PNC with a skilled health provider. All measures were coded as 0 for “no” or 1 for “yes”. The definition of ANC visits refers to the WHO Focused ANC (FANC) and the Ministry of Health Decree No.97/2014, which includes the first visit in the first trimester, second visit at second trimester, and the third and fourth visits at third trimester [28]. We further assessed the clinical intervention received by respondents: including weight and height measurement, blood pressure measurement, urine and blood sample taken, stomach examined, consultation, and iron supplementation. Women who had ANC4+ and received the eight essential clinical components of routine ANC from skilled providers were categorised receiving good quality of ANC services. Skilled birth attendance was defined as delivery attended by doctor, nurse, midwife, and village/auxiliary midwife [29]. Facility-based delivery was defined as delivery at a hospital, health centre, maternity home, and clinic. PNC was defined as receiving care within 2 days after delivery (detailed definitions in Table S1 in the **Online Supplementary Document**) [26].

#### Treatment and control groups

The primary independent variable is the respondent’s enrolment in JKN, using the questions: “Are you covered by any health insurance?” and “What type of health insurance are you covered by?” to categorise the following:

- Treatment group (N = 3,661): respondents who answered affirmatively for having JKN coverage (either PBI/Jamkesda (regional health insurance) or Non-PBI).
- Control group (N = 2,044): respondents who had no health insurance at the time of the survey.

#### Covariates

We controlled for maternal age at the time of the survey, marital status, birth order, level of education, women’s employment status, wealth index (as constructed by the DHS based on household assets, housing materials, water access and sanitation facilities), media exposure (internet and newspaper), residency and region of residence [26]. Wealth index and region of residence were used to measure inequalities in access to maternal health services. Existing studies were used in selecting covariates to minimise selection bias of JKN enrolment [16,23,24,30].

### Statistical analysis

Propensity scoring matching (PSM) approach was applied to compare maternal health services utilisation among those insured and not insured with JKN. This statistical method was selected as women’s enrolment in health insurance is not random and can be strongly influenced by unobservable and observable characteristics (selection bias) [23,24]. In brief, the analysis consists of three stages.

*First*, we applied a logit model (due to the dichotomous outcome indicator) to estimate the propensity score, or a predicted probability of insurance by JKN given a set of observable covariates. *Second,* we used the *psmatch2* command to match participants and non-participants on the basis of the propensity score. To ensure that the propensity score’s distribution was similar for insured (treatment) and uninsured (control) women, we evaluate the quality of matched pairs by applying balance diagnostic test. Following the Rosenbaum and Rubin, we used absolute standardised mean difference as the most common measure to ensure balance within treatment and control group with respect to the covariates [31-33]. Studies indicates that standardised mean differences less than 10% is considered balance [31,34]. We also computed other metrics, pseudo-R^2^, Rubin’s *B* and Rubin’s *R*, as additional measures to ensure our quality of matching. The Rubin’s *B* should be less than 25 and *R* ranged from 0.5 to 2. The balance check was performed for different matching algorithms, including nearest neighbour (NN) with and without replacement, radius matching, and Kernel matching. We selected Kernel matching for our main analysis as the result shows the satisfactory balance in all diagnostics checks (balancing check in Table S2-S4 in the **Online Supplementary Document**). *Lastly,* we use the *kmatch* command [35] to estimate the difference in maternal health services utilisation between the treated and control with a calliper of bandwidth equal to 0.2 of the standard deviation of the logit of the propensity score. To improve accuracy, bootstrapping with 200 replications was performed to estimate the standard error. Stratified analyses were conducted to assess the impact of JKN across population groups. All statistical analyses were conducted using Stata 14.2 SE (StataCorp, College Station, Texas 77845, USA).

### Robustness check

We performed a sensitivity analysis using Coarsened Exact Matching (CEM) algorithm. CEM matched the treatment group to the control group with the exact same covariates value, which mostly produces very few matches and may also narrow the representativeness of the remaining sample [36]. We also conducted a sensitivity analysis using the Mantel and Haenszel (MH) method. The Gamma coefficient (Γ) = 1 indicates there is no hidden bias, while higher values of Γ indicates more influence of unobserved factors. This study set the Γ between 1 and 2 with an increment of 0.1, an approach adopted by previous studies using PSM [23,37]. Furthermore, we applied the multiple hypothesis testing using Bonferroni and Holm procedure to control Type 1 error (the probability of rejecting a true null hypothesis) inflation due to simultaneous testing of null hypotheses for the six outcomes [38,39].

Any public health insurance programme, financing strategies or otherwise, can have unintended consequences. We investigated the potential negative impact of JKN on access to maternal health services for the uninsured population comparing their health care service utilisation rates before and after the implementation of JKN, using data from 2012 to 2017.

## RESULTS

### Sample characteristics

The characteristics of our study sample are reported in **Table 1**. Overall, 61% of the women who recently gave birth were insured by JKN in 2017. The majority of respondents were less than 35 years old, married, with two or fewer children, had completed secondary education, had no occupation, were not exposed to internet and newspaper, and lived in Java-Bali region. Despite the poor and near-poor being eligible for full or partial subsidies through JKN, there was little difference in the distribution of respondents across wealth quintiles within the insured and uninsured population, with the exception being fewer respondents within the highest wealth quintile among the uninsured group. The proportion of women receiving maternal health services was higher for those insured with JKN compared with the uninsured: (1) ANC4+ visits (78.6% vs 71.0%); (2) ANC 4+ visits and received clinical components of ANC (23.4% vs 19.8%); (3) SBA (94.4% vs 90.6%); (4) facility-based delivery (86.8% vs 78.1%); (5) PNC (90.2% vs 85.4%); (6) PNC with skilled providers (89.3% vs 83.7%). Sample characteristics for 2012 IDHS are available in Table S3 in the **Online Supplementary Document**.

**Table 1:** Background characteristics of women who had recent live birth between 2016 and 2017*

Variables	All		Treatment: insured by JKN		Control: uninsured by JKN	
%	N	%	n	%	n
**Overall†**	100.0	5429	61.4	3332	38.3	2097
**Outcomes variables:**						
(a) ANC 4+	75.6	4107	78.6	2618	71.0	1489
(b) ANC 4+ and received clinical components of ANC	22.0	1194	23.4	780	19.8	414
(c) Skilled birth attendance	92.9	5045	94.4	2892	90.6	1900
(d) Facility-based delivery	83.5	4531	86.8	3145	78.1	1639
(e) PNC	88.4	4795	90.2	3004	85.4	1791
(f) PNC with skilled provider	87.1	4729	89.3	2097	83.7	1756
**Control variables:**						
Age (in years):						
15-24	25.1	1364	22.9	764	28.6	600
25-34	51.9	2819	52.4	1745	51.2	1074
35-42	21.0	1140	22.4	747	18.7	392
42-49	2.0	107	2.3	76	1.5	31
Marital status:						
Unmarried	1.9	104	1.6	54	2.4	50
Married	98.1	5325	98.4	3277	97.6	2047
**Birth order:**						
1	32.7	1776	32.4	1081	33.2	695
2	35.3	1918	34.2	1139	37.1	778
3	18.9	1027	19.5	648	18.1	379
4	8.0	437	8.4	279	7.5	158
5 or more	5.0	271	5.5	184	4.2	87
**Education:**						
None/incomplete primary	6.5	352	6.4	212	6.7	140
Complete primary	18.0	979	16.9	562	19.9	417
Incomplete secondary	28.3	1535	25.6	852	32.6	683
Complete secondary	31.0	1684	30.9	1029	31.2	655
Higher/vocational	16.2	879	20.3	677	9.6	202
**Employment:**						
None	56.7	3076	52.7	1755	63	1320
Agriculture	6.8	367	6.2	206	7.6	160
Blue-collar	24.9	1352	25.5	849	24	503
White-collar	11.7	634	15.6	520	5.4	114
**Exposure to internet:**						
Not at all	61.7	3351	57.5	1916	68.4	1435
Less than once a week	30.3	1645	32.7	1090	26.5	556
At least once a week	8.0	433	9.8	326	5.1	107
**Exposure to newspaper:**						
Not at all	4.1	220	3.8	128	4.4	92
Less than once a week	12.9	702	13.3	443	12.3	259
At least once a week	83.0	4506	82.9	2760	83.3	1746
**Wealth index:**						
Very poor	20.2	1098	19.5	650	21.4	448
Poor	20.9	1135	19.2	640	23.6	495
Middle	19.9	1079	19.2	640	20.9	439
Rich	20.8	1129	20.6	688	21.1	442
Very rich	18.2	987	21.4	714	13.0	273
**Residency:**						
Rural	51.9	2818	48.3	1610	57.6	1208
Urban	48.1	2611	51.7	1721	42.4	890
**Region of residency:**						
Eastern Indonesia	3.5	192	3.9	131	2.9	62
Sulawesi	7.4	402	8.9	297	5	105
Kalimantan	6.0	326	5.2	174	7.2	152
Nusa Tenggara	4.9	263	4.5	151	5.4	113
Sumatra	22.7	1235	22.9	765	22.4	470
Java & Bali	55.4	3010	54.4	1814	57	1196

PNC – post-natal care, JKN – *Jaminan Kesehatan Nasional*

*This table includes all women who had birth recent live birth between 2016–2017 before matching. ANC 4+: At least 4 antenatal care visits. Percentages and Ns are weighted. Unweighted sample size = 5717.

### Propensity score estimation and balance diagnostics test

**Table 2** presents the predicted probability of enrolment in JKN according to logistic regression model. Type of employment, exposure to internet, and residency were the most important predictors of women’s enrolment status in JKN. For example, women who work in white-collar jobs were 2.34 times (95% CI = 1.74-3.16) more likely to be insured by JKN compared to women who had no occupation. Women who were exposed to internet at least once a week had 1.46 (95% CI = 1.09-1.97) higher probability of being insured in JKN than women who did not expose to internet. Those who lived in urban area had 1.36 (95% CI = 1.15-1.62) higher odds of being insured by JKN compared to rural groups. No significant differences in enrolment status were found for the other wealth quintiles, as compared to the poorest group **(****Table 2**).

**Table 2:** Factor associated with *Jaminan Kesehatan Nasional* (JKN) enrolment in 2017*

Variables	AOR	(95% CI)	*P*-value
Age:			
15–24 y	Ref.		
25–34 y	1.12	(0.92-1.37)	0.248
35–42 y	1.31	(1.00-1.73)	0.054
42–49 y	1.76	(0.95-3.26)	0.073
**Marital status:**			
Unmarried	Ref.		
Married	1.37	(0.86-2.18)	0.187
Birth order:			
1	Ref.		
2	0.96	(0.79-1.17)	0.695
3	1.06	(0.83-1.36)	0.655
4	1.02	(0.74-1.39)	0.915
5 or more	1.25	(0.83-1.87)	0.284
Education:			
None/incomplete primary	Ref.		
Complete primary	0.94	(0.70-1.27)	0.684
Incomplete secondary	0.89	(0.66-1.20)	0.461
Complete secondary	0.97	(0.71-1.33)	0.855
Higher/vocational	1.25	(0.86-1.80)	0.238
Employment:			
None	Ref.		
Agriculture	0.96	(0.74-1.27)	0.794
Blue-collar	1.17	(0.99-1.40)	0.073
White-collar	2.34	(1.74-3.16)	<0.0001
**Exposure to internet:**			
Not at all	Ref.		
Less than once a week	1.26	(1.07-1.47)	0.004
At least once a week	1.46	(1.09-1.97)	0.012
**Exposure to newspaper:**			
Not at all	Ref.		
Less than once a week	1.10	(0.76-1.58)	0.615
At least once a week	1.05	(0.75-1.48)	0.781
**Wealth index:**			
Very poor	Ref.		
Poor	0.85	(0.67-1.08)	0.173
Middle	0.89	(0.68-1.15)	0.365
Rich	0.82	(0.62-1.08)	0.150
Very rich	1.04	(0.75-1.44)	0.812
**Residency:**			
Rural	Ref.		
Urban	1.36	(1.15-1.62)	<0.0001
**Region of residency:**			
Eastern Indonesia	Ref.		
Sulawesi	1.28	(0.92-1.80)	0.148
Kalimantan	0.55	(0.38-0.78)	0.001
Nusa Tenggara	0.63	(0.44-0.90)	0.011
Sumatra	0.73	(0.53-1.01)	0.059
Java & Bali	0.68	(0.49-0.94)	0.021

AOR – adjusted odds ratio, CI – confidence interval

*Hosmer and Lemeshow Test of goodness of fit: *P*-value = 0.959.

According to the balance diagnostic checks, we verified that Kernel matching resulted satisfactory balance for all model parameters (in Table S2-S3 in the **Online Supplementary Document**). After matching, the treatment and control group became comparable, as shown by low standardised difference in means across all covariates (<10%). We also estimated the low pseudo-R2, with Rubin’s B and Rubin’s R were 7.4 and 0.92, respectively. The distribution of the propensity score, including region of common support, by control and treatment groups is presented in Figure S2 in the **Online Supplementary Document**. A total of 12 treated participants were off support after matching and were removed from the sample. The result of the balancing test for the other matching algorithms is available in Table S4 in the **Online Supplementary Document**.

### Effects of JKN on maternal health outcomes

Table 3 shows the difference in maternal health services utilisation between the insured and uninsured at the national level, as measured by the average treatment effect on the treated (ATT). Enrollment in JKN was associated with an improvement in receiving all six maternal health services (*P* < 0.0001). The highest effect size was found for facility-based delivery, while the smallest effect was for SBA. Enrollment in JKN was associated with greater prevalence in ANC 4+ visits by 7.4% (95% CI = 4.8-9.9), ANC 4+ visits and received clinical components of routine ANC by 5.6% (95% CI = 3.3-7.9), skilled birth attendance by 3.0% (95% CI = 1.5-4.5), facility-based delivery by 10.2 (95% CI = 7.5-12.7), PNC by 4% (95% CI = 2.2-5.7), and PNC with skilled provider by 4.5% (95% CI = 2.6-6.5). **Table 3** also shows the percentage change in outcome variables relative to the control group.

**Table 3:** The average treatment effect on treated (ATT) of *Jaminan Kesehatan Nasional* (JKN) on maternal health services*

Variables		Treatment group: Insured by JKN	Control group: Uninsured by JKN	ATT†		Percentage changes	SE
		**%**	**%**	**%**	**(95% CI) †**	**%**	
(a) % At least 4 ANC visits	Unmatched	73.0	65.2	7.8			1.2
	Matched	73.1	65.7	7.4	(4.8-9.9)***	8.8%	1.5
(b) % At least 4 ANC visits and received clinical components of ANC	Unmatched	21.0	16.3	4.7			1.1
	Matched	21.0	15.4	5.6	(3.3-7.9)***	36.4%	1.2
(c) % Skilled birth attendance	Unmatched	92.7	88.0	4.7			0.8
	Matched	92.7	89.7	3.0	(1.5-4.5)***	3.3%	0.9
(d) % Facility-based delivery	Unmatched	82.2	70.0	12.2			1.1
	Matched	82.2	72.0	10.2	(7.5-12.7)***	14.2%	1.4
(e) % PNC	Unmatched	88.4	81.9	6.4			09
	Matched	88.4	84.4	4.0	(2.2-5.7)***	4.7%	1.1
(f) % PNC with skilled providers	Unmatched	87.1	79.8	7.3			0.8
	Matched	87.1	82.5	4.5	(2.6-6.5)***	5.5%	1.0

ANC – antenatal care, PNC – post-natal care, SE – standard error, ATT – treatment effect on the treated, CI – confidence interval

*N after matching = 5705

†The average treatment effect on the treated (ATT) was calculated using psmatch2 command to estimate the difference of maternal care utilisation between treatment and control. We used Kernel matching with a calliper of bandwidth equal to 0.2 of the standard deviation of the logit of propensity score. To improve accuracy, bootstrapping with 200 times replications was performed to estimate the standard error.

‡Significance: *P < 0.05; **P < 0.01; ***P < 0.001.

### Differential effect of JKN by sociodemographic groups

#### Economic groups

**Figure 1:**
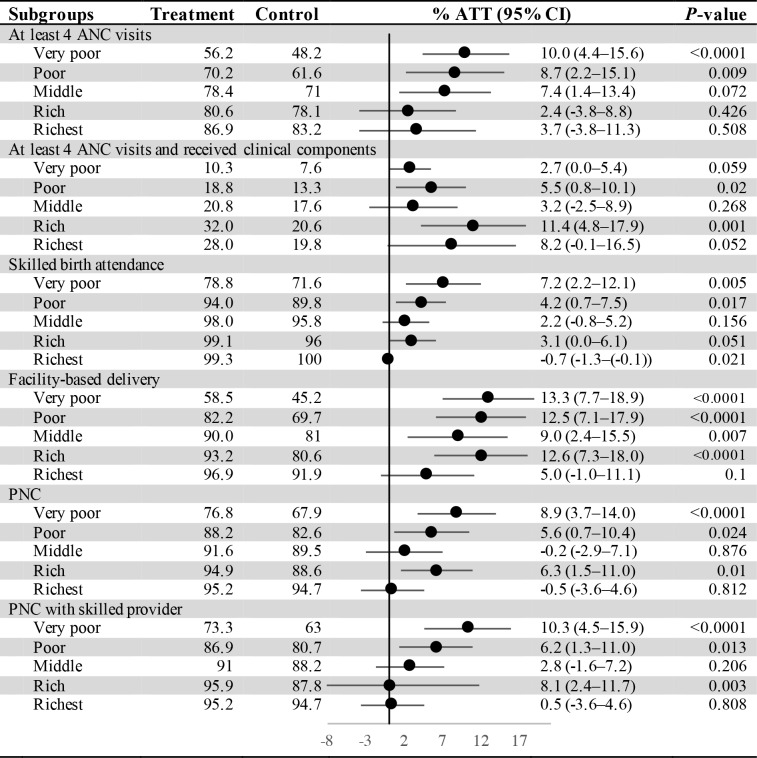
The average treatment effect on treated (ATT) of *Jaminan Kesehatan Nasional* (JKN) on maternal health services, by economic status. ANC – antenatal care, PNC – post-natal care.

Our results by economic subgroup (**Figure 1**) indicate that the differences in outcomes associated with JKN enrollment were larger for the poorest wealth quintiles compared to the most affluent, although the 95% confidence intervals overlapped in all but the skilled birth attendance outcome (ATT = 7.2, 95% CI = 2.2-12.1 vs ATT = -0.7 95% CI = -1.3to -0.1). However, the effect of JKN enrollment on the quality of routine ANC visits was more pronounced in the rich wealth quantiles. Substantial gaps in maternal health services utilization between the poorest and richest wealth quintiles still exist. For instance, facility-based delivery was 38 percentage points lower for the poorest quintile (58.5%) compared to the richest (96.9%) among those insured by JKN.

#### Regional areas

**Figure 2:**
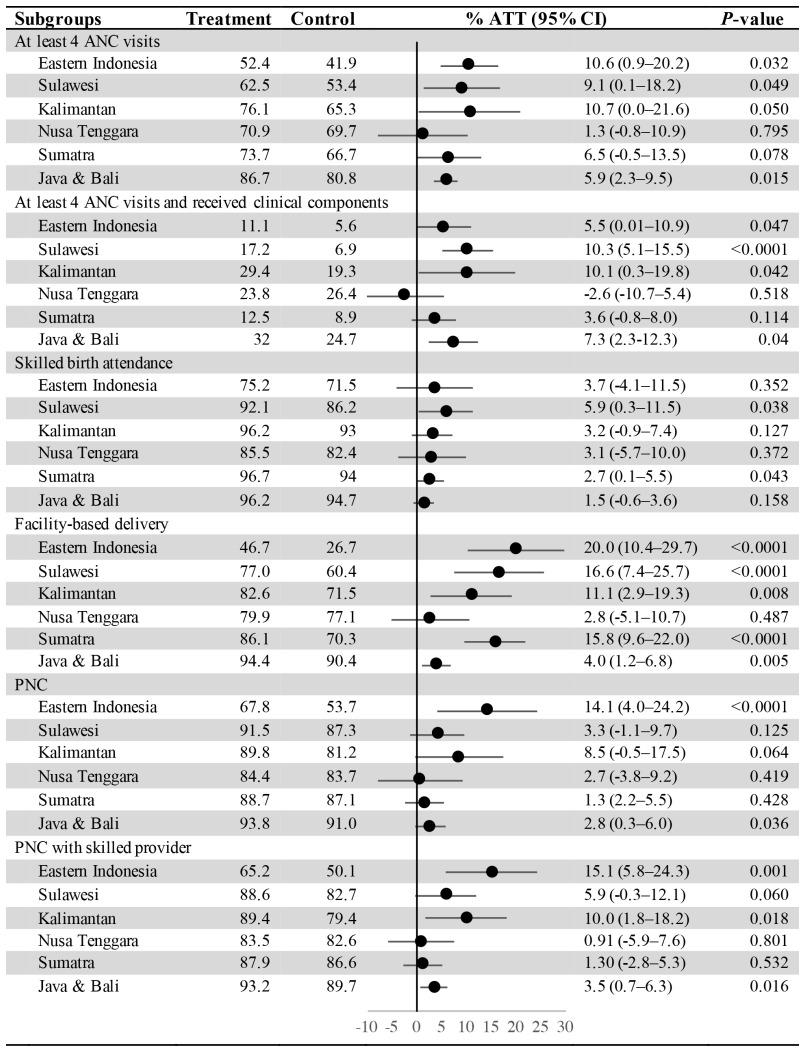
The average treatment effect on treated (ATT) of *Jaminan Kesehatan Nasional* (JKN) on maternal health services, by regional of residency. ANC – antenatal care, PNC – post-natal care.

**Figure 2** shows the difference in maternal health services utilisation between the insured and uninsured across regional areas. Our results indicate that JKN was associated with greater utilisation in Eastern Indonesia compared to Java & Bali for three outcomes: (1) facility-based delivery (ATT = 20, 95% CI = 10.4-29.7 vs ATT = 4.0, 95% CI = 1.2-6.8), PNC (ATT = 14.1, 95% CI = 4.0-24.2 vs ATT = 2.8, 95% CI = 0.3-6.0), and PNC with skilled providers (ATT = 15.1, 95% CI = 5.8-24.3 vs ATT = 3.5, 95% CI = 0.7-6.3). Large and statistically significant differences in ATT were also found for Sulawesi and Kalimantan, for some of utilisation outcomes. However, the findings indicate that inequalities still persist across regional areas. Utilisation of all maternal health services in Eastern Indonesia was much lower than in Sumatra and Java & Bali. For instance, among those insured by JKN, the percentage of ANC4+ visits in Eastern Indonesia (52.4%) was 34.3 percentage points lower than in Java & Bali (86.7%).

### Unintended consequences of JKN

**Figure 3:**
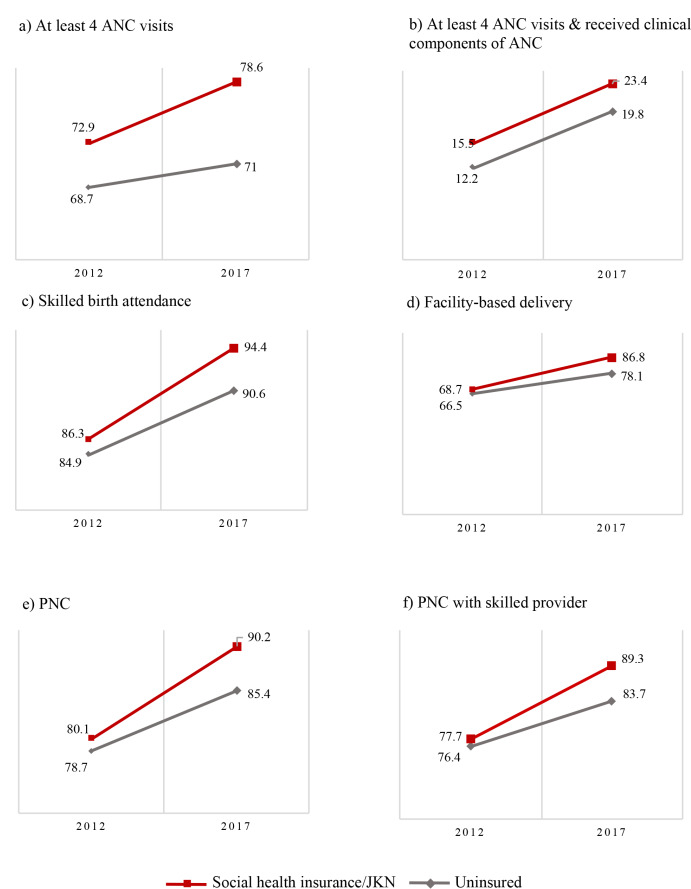
Trend of maternal health services by health insurance enrolment, Indonesia 2012-2017. We only included women who had recent live birth in 2011-2012 (2012 IDHS) and 2016-2017 (2017 IDHS). Number of samples in 2012 = 4432. Number of samples in 2017 = 5429. Percentages and numbers are weighted.

We tested whether there were unintended negative impacts of JKN for uninsured women. According to **Figure 3**, the trend of maternal service utilisation increased for both the insured and uninsured from 2012 to 2017. The trend increased for ANC4+ (68.7% vs 71.0%); ANC4+ with skilled providers (12.2% vs19.8%); SBA (84.9% vs 90.6%); facility-based delivery (66.5% vs 78.1%); PNC (78.7% vs 90.9%); and PNC with skilled providers (76.4% vs 83.7%) among the uninsured. Therefore, it appears that JKN was not associated with a reduction on utilisation among the uninsured.

### Sensitivity analysis and robustness check

All matching algorithms show consistent results that implementation of JKN was associated with an improvement in access to maternal health services. NN with replacement had a higher ATT compared to others, but has a poorer quality of matching covariates (in Table S6 in the **Online Supplementary Document**). Using CEM reduced the sample size to 1657 observations or removed approximately 70% of observations compared to Kernel matching (N = 5705). CEM results suggest a slightly lower effect of JKN compared to Kernel matching across six outcomes (in Table S8 in the **Online Supplementary Document**). However, no significant effect was observed in skilled birth attendance outcome. The results of sub-group analysis also demonstrate that the benefit of JKN appears more pronounced among poor groups. However, JKN is mostly not statistically significant in regional areas subgroup analysis, most likely due to the small sample sizes covariates (in Table S9-S10 in the **Online Supplementary Document**). Finally, the results of the MH test imply that the results are moderately robust and insensitive to hidden bias at Γ = 1.5 in almost all outcome variables (in Table S11 in **Online Supplementary Document**). *P*-values obtained by multiple hypothesis testing indicates that association between JKN and maternal health service use is still significant at the 5% significance level (in Table S12 in the **Online Supplementary Document**).

## DISCUSSION

Our findings show that women insured by JKN have improved access to maternal health care services along the full continuum of care, compared with uninsured women. Our study also shows that these findings were more pronounced among women in the poorest quintile households and those residing in Eastern Indonesia, except for the quality of ANC visits. It is important to note that maternal health services coverage rates among higher wealth quintiles and in more developed regions are generally very high, and as such have less potential for increase as compared to lower coverage rates among the lower wealth quintiles and less developed regions. Thus, our results suggest that there is a greater potential for improvements when the coverage of maternal health services is low, for example, in lower-income groups and less developed regions. However, the findings indicate that JKN is far from closing the gap in access between the poor and non-poor: inequalities still persist in the coverage of maternal health services across socioeconomic groups and geographical areas of Indonesia. These inequalities imply an inequitable distribution of the subsidy and maternal health services provided within JKN. While a full benefit incidence analysis is beyond the scope of this paper, clearly JKN funding follows utilisation, and is concentrated where utilisation is highest in absolute terms. More remains to be done in supporting improved access to services in marginalised population groups.

Our findings are consistent with previous studies from Indonesia and other LMICs [20,22,23,30,40-42]. Most studies have found health insurance to be significantly associated with an increased use of health care services. Despite these findings, our study is consistent with others in finding that JKN has not closed the gap of access to services within regions, in particular in Eastern Indonesia, where the availability of human resources for health and facilities is still much lower compared to other regions [16,20,43]. In India, a conditional cash transfer program was found to have a greater effect on maternal health service use in worse-off states with low coverage of maternal health services, compared to better-off states [41,44].

Despite this study’s promising findings, our findings suggested that the impact of JKN on quality of ANC was more pronounced among the higher socio-economic groups. Poor supply-side readiness and inadequate quality of care remain important challenges in Indonesia [9,12,45-47]. The referral system is still lacking, resulting in delays in service provision. Necessary supplies and equipment, such as blood transfusion, may not always be available [47]. These challenges are particularly evident in East Kalimantan, a province with the highest rates of facility-based delivery and SBA in Kalimantan but persistently high MMR [46]. The 2016 Indonesia Quantitative Service Delivery Survey revealed that the ability of health providers to deliver ANC, identify risk of complications, and manage complications according to guidelines is still weak [9]. While this study used limited measures of quality of care, it is clear that expanding coverage of health insurance should be done simultaneously with improving supply-side readiness, and progress towards UHC should also be measured through the lens of service quality [48].

WHO Strategies toward Ending Preventable Maternal Mortality seeks to “ensure universal health coverage for comprehensive sexual, reproductive, maternal and newborn health” [2]. Based on current trends, Indonesia failed to achieve UHC by 2019 and may only achieve this target by 2034 [13,19]. One of the obstacles to achieving UHC is reaching the ‘missing middle population' or those who work in the informal sector with lower-middle income [12,13,43,49]. In 2016, only 7% of this population was covered by JKN [49]. Since they must pay insurance premiums themselves, their willingness-to-pay becomes an important underlying factor that determines their enrolment. The poor availability and accessibility to health care services, as well as lack of understanding on the significance of health insurance are the main reasons that could impede their participation in the JKN [12,50]. Efforts should be made to reduce the disparities in the availability of health services in less-developed regions and raise public awareness about the importance of health insurance, in addition to the use of sanctions for individuals and employers [12,51].

Another major problem is a potential mistargeting of the poor and non-poor [12,49]. Despite eligibility rules for full or partially subsidized premiums for the very poor and near-poor, around 40.8% of the very poor households in our sample were not covered by JKN in 2017. A report published by World Bank produced a similar result: around half of very poor households, which should be categorized as subsidized participants, were uninsured by JKN in 2016 [49]. BPJS-K and Ministry of Social Affairs should collaborate with local government to validate the lists of poor and near-poor households and eliminate mistargeting subsidised beneficiaries.

This study has several important limitations. First, enrolment status in JKN was asked at the time of the interview, not at the time of pregnancy and delivery. We assumed that women had the same enrolment status at the time of interview and during their previous pregnancy. We attempted to address this timing issue by only including women who had had a recent live birth between 2016-2017, a year preceding the survey, as others have done [30,40,42]. Second, although PSM can reduce the selection bias of JKN enrolment, the analysis was cross-sectional and can only demonstrate the association between the variables, not causality. It is plausible that those who have opportunities to use maternal services have more motivation to enrol in JKN than those who do not, for example because they live distant from available services. Third, the analysis may still be subject to the problem of unobservable characteristics. For example, we have not included controls for religion or ethnicity, which have been found to be associated with utilisation of maternal health services [16,30,42], but was not available in the data set. We attempted to minimise and test for the potential for bias through sensitivity analyses. Different matching techniques and tests for unobserved confounding indicate these results are robust and insensitive to bias. Fourth, sample sizes for some of the regional sub-group analyses were small, resulting in insignificant results with very wide confidence intervals. Fifth, we only conducted pre- and post-analysis using serial cross-sectional data from 2012 and 2017, which may not reflect the current pattern. Our trend analysis may also be limited by issues of selection bias and unobservable confounding as the uninsured population pre-JKN may not be comparable with the uninsured post-JKN. Further analyses are needed to explore this further with longitudinal data if possible. Despite these limitations, our study offers compelling evidence on the positive association of health insurance on utilisation of care at a national level, and sub-nationally by income group and regional area.

## CONCLUSIONS

This is the first study assessing the effect of JKN on access to maternal health services across the continuum of care, using the most recent nationally representative data. Evidence from this study reveals that government efforts to eliminate financial barriers and provide equitable access through national health insurance can lead to improved access to maternal health services, especially for disadvantaged groups. However, there remain significant socioeconomic and regional inequality in access to maternal health services in Indonesia. Continued health system reforms in Indonesia must place greater emphasis on providing improved access to those disadvantaged groups.

## Additional material

Online Supplementary Document

## References

[1] World Health Organization. Maternal mortality: key facts 2018. Available: https://www.who.int/news-room/fact-sheets/detail/maternal-mortality. Accessed: 30 April 2019.

[2] World Health Organization. Strategies toward ending preventable maternal mortality (EPMM). Geneva: WHO, 2015.

[3] GBD 2015 Maternal Mortality Collaborators Global, regional, and national levels of maternal mortality, 1990-2015: a systematic analysis for the Global Burden of Disease Study 2015. Lancet. 2016;388:1775-812. 10.1016/S0140-6736(16)31470-2 27733286 PMC5224694

[4] The Association of Southeast Asian Nations (ASEAN) Secretariat. ASEAN Statistical Report on Millennium Development Goals 2017. Jakarta: 2017.

[5] Statistics Indonesia (BPS). 2015 Intercensal Population Survey. Jakarta: BPS, 2015.

[6] Statistics Indonesia. Maternal mortality ratio by island. 2015 Intercensal Popul Surv. 2018. Available: https://www.bps.go.id/dynamictable/2018/06/05/1439/angka-kematian-ibu-menurut-pulau-per-100-000-kelahiran-hidup-2015.html. Accessed: 10 March 2019.

[7] Knaul FM Langer A Atun R Rodin D Frenk J Bonita R Rethinking maternal health. Lancet Glob Health. 2016;4:e227-8. 10.1016/S2214-109X(16)00044-9 26953968

[8] Miller S Belizán JM The true cost of maternal death: Individual tragedy impacts family, community and nations. Reprod Health. 2015;12:56. 10.1186/s12978-015-0046-3 26081494 PMC4470047

[9] Yap WA, Pembudi ES, Marzoeki P, Cain JS, Tandon A. Revealing the missing link: Private sector supply-side readiness for primary maternal health services in Indonesia. Jakarta: World Bank, 2017.

[10] World Health Organization. State of inequality: reproductive, maternal, newborn, and child health. Geneva: WHO; 2015.

[11] Belton S Myers B Ngana FR Maternal deaths in eastern Indonesia: 20 years and still walking: an ethnographic study. BMC Pregnancy Childbirth. 2014;14:39. 10.1186/1471-2393-14-39 24447873 PMC3901769

[12] Agustina R Dartanto T Sitompul R Susiloretni KA Achadi ELet al. Universal health coverage in Indonesia: concept, progress, and challenges. Lancet. 2019;393:75-102. 10.1016/S0140-6736(18)31647-7 30579611

[13] Wiseman V Thabrany H Asante A Haemmerli M Kosen S Gilson L An evaluation of health systems equity in Indonesia: study protocol. Int J Equity Health. 2018;17:138. 10.1186/s12939-018-0822-0 30208921 PMC6134712

[14] National Research Council. Reducing Maternal and Neonatal Mortality in Indonesia: Saving Lives, Saving the Future. Washington, DC: The National Academies Press; 2013. 24851304

[15] World Health Organization and International Bank for Reconstruction and Development / The World Bank. Tracking universal health coverage: 2017 global monitoring report. 2017.

[16] Nababan HY Hasan M Marthias T Dhital R Rahman A Anwar I Trends and inequities in use of maternal health care services in Indonesia, 1986-2012. Int J Womens Health. 2017;10:11-24. 10.2147/IJWH.S144828 29343991 PMC5749568

[17] Mboi N Murty Surbakti I Trihandini I Elyazar I Houston Smith K Bahjuri Ali P On the road to universal health care in Indonesia, 1990–2016: a systematic analysis for the Global Burden of Disease Study 2016. Lancet. 2018;392:581-91. 10.1016/S0140-6736(18)30595-6 29961639 PMC6099123

[18] Social Security Agency for Health. Total participants of JKN 2019. Available: https://bpjs-kesehatan.go.id/bpjs/. Accessed: 27 December 27 2019).

[19] Defisit BPJS Kesehatan Belum Akan BerakhirSuara Pembaruan 2018:3.

[20] Teplitskaya L, Dutta A. Has Indonesia’s National Health Insurance Scheme Improved Access to Maternal and Newborn Health Services? Washington, DC: Palladium, Health Policy Plus, 2018.

[21] Global Burden of Disease Health Financing Collaborator Network Past, present, and future of global health financing: a review of development assistance, government, out-of-pocket, and other private spending on health for 195 countries, 1995–2050. Lancet. 2019;393:2233-60. 10.1016/S0140-6736(19)30841-4 31030984 PMC6548764

[22] Brooks MI Thabrany H Fox MP Wirtz VJ Feeley FG Sabin LL Health facility and skilled birth deliveries among poor women with Jamkesmas health insurance in Indonesia: a mixed-methods study. BMC Health Serv Res. 2017;17:105. 10.1186/s12913-017-2028-3 28148258 PMC5288898

[23] Wang W Temsah G Mallick L The impact of health insurance on maternal health care utilization: evidence from Ghana, Indonesia and Rwanda. Health Policy Plan. 2017;32:366-75. 28365754 10.1093/heapol/czw135 PMC5400062

[24] Erlangga D Ali S Bloor K The impact of public health insurance on healthcare utilisation in Indonesia: evidence from panel data. Int J Public Health. 2019;64:603-13. 10.1007/s00038-019-01215-2 30737522 PMC6517357

[25] von Elm E Altman DG Egger M Pocock SJ Gøtzsche PC Vandenbroucke JP The Strengthening the Reporting of Observational Studies in Epidemiology (STROBE) statement: guidelines for reporting observational studies. Lancet. 2007;370:1453-7. 10.1016/S0140-6736(07)61602-X 18064739

[26] National Population and Family Planning Board (BKKBN), Statistics Indonesia (BPS), Ministry of Health. (Kemenkes), and ICF. Indonesia demographic and health survey 2017. Jakarta: BKKBN, BPS, Kemenkes, and ICF, 2018.

[27] Statistics Indonesia (BPS), National Population and Family Planning Board (BKKBN), Ministry of Health. (Kemenkes), and ICF International. Indonesia demographic and health survey 2012. Jakarta: BPS, BKKBN, Kemenkes, and ICF International, 2013.

[28] Minister of Health of the Republic of Indonesia. Ministry of Health Decree no 97/2014 on Pre-natal, Pregnancy, Postnatal, Sexual and Reproductive Health Services. 2014.

[29] World Health Organization. Skilled birth attendants 2014. Available: https://www.who.int/reproductivehealth/topics/mdgs/skilled_birth_attendant/en/. Accessed: 14 May 14 2019.

[30] Bonfrer I Breebaart L Van de Poel E The effects of Ghana’s national health insurance scheme on maternal and infant health care utilization. PLoS One. 2016;11:e0165623. 10.1371/journal.pone.0165623 27835639 PMC5106190

[31] Stuart EA Lee BK Leacy FP Prognostic score-based balance measures can be a useful diagnostic for propensity score methods in comparative effectiveness research. J Clin Epidemiol. 2013;66:S84-S90. 10.1016/j.jclinepi.2013.01.013 23849158 PMC3713509

[32] Ali MS Groenwold RHH Klungel OH Best (but oft-forgotten) practices: Propensity score methods in clinical nutrition research. Am J Clin Nutr. 2016;104:247-58. 10.3945/ajcn.115.125914 27413128

[33] Rubin DB Using Propensity Scores to Help Design Observational Studies: Application to the Tobacco Litigation. Health Serv Outcomes Res Methodol. 2001;2:169-88. 10.1023/A:1020363010465

[34] Ravit M Ravalihasy A Audibert M Ridde V Bonnet E Raffalli B The impact of the obstetrical risk insurance scheme in Mauritania on maternal healthcare utilization: a propensity score matching analysis. Health Policy Plan. 2020;35:388-98. 10.1093/heapol/czz150 32003810 PMC7195851

[35] Jann B. KMATCH: Stata module module for multivariate-distance and propensity-score matching, including entropy balancing, inverse probability weighting, (coarsened) exact matching, and regression adjustment 2017. Available: https://ideas.repec.org/c/boc/bocode/s458346.html. Accessed: 10 February 2019.

[36] Blackwell M, Iacus S, King G, Porro G. Coarsened Exact Matching in Stata. 2010.

[37] Becker SO Caliendo M Sensitivity analysis for average treatment effects. Stata J. 2007;7:71-83. 10.1177/1536867X0700700104

[38] Streiner DL Best (but oft-forgotten) practices: the multiple problems of multiplicity—whether and how to correct for many statistical tests. Am J Clin Nutr. 2015;102:721-8. 10.3945/ajcn.115.113548 26245806

[39] Newson R Multiple-test procedures and smile plots. Stata J. 2003;3:109-32. 10.1177/1536867X0300300202

[40] Mensah J Oppong JR Schmidt CM Ghana’s national health insurance scheme in the context of the health MDGs: an empirical evaluation using propensity score matching. Health Econ. 2010;19:95-106. 10.1002/hec.1633 20730999

[41] Lim SS Dandona L Hoisington JA James SL Hogan MC Gakidou E India’s Janani Suraksha Yojana, a conditional cash transfer programme to increase births in health facilities: an impact evaluation. Lancet. 2010;375:2009-23. 10.1016/S0140-6736(10)60744-1 20569841

[42] Gouda HN Hodge A Bermejo R Zeck W Jimenez-Soto E The impact of healthcare insurance on the utilisation of facility-based delivery for childbirth in the Philippines. PLoS One. 2016;11:e0167268. 10.1371/journal.pone.0167268 27911935 PMC5135090

[43] Hartwig R Sparrow R Budiyati S Yumna A Warda N Suryahadi A Effects of decentralized health-care financing on maternal care in Indonesia. Econ Dev Cult Change. 2019;67:659-86. 10.1086/698312

[44] Carvalho N Rokicki S The impact of India’s Janani Suraksha Yojana Conditional Cash Transfer Programme: A replication study. J Dev Stud. 2019;55:989-1006. 10.1080/00220388.2018.1506578

[45] Yogesh R, Jessica G, Iva D, Sayaka K, Martha C, Kebba J, et al. Re-envisioning maternal and newborn health in Indonesia: how the private sector and civil society can ignite change. Washington, DC: Palladium, Health Policy Plus, 2016.

[46] Mahmood MA Mufidah I Scroggs S Siddiqui AR Raheel H Wibdarminto K Root-cause analysis of persistently high maternal mortality in a rural district of Indonesia: Role of clinical care quality and health services organizational factors. Biomed Res Int. 2018;2018. 10.1155/2018/3673265 29682538 PMC5842724

[47] Rosales A Sulistyo S Miko O Hairani LK Ilyana M Thomas J Recognition of and care-seeking for maternal and newborn complications in Jayawijaya district, Papua province, Indonesia: a qualitative study. J Health Popul Nutr. 2017;36:44. 10.1186/s41043-017-0122-0 29297380 PMC5764054

[48] Kruk ME Gage AD Arsenault C Jordan K Leslie HH Roder-DeWan S High-quality health systems in the Sustainable Development Goals era: time for a revolution. Lancet Glob Health. 2018;6:e1196-252. 10.1016/S2214-109X(18)30386-3 30196093 PMC7734391

[49] Tandon A, Pambudi E, Harimurti P, Masaki E, et al. Indonesia Health Financing System Assessment Spend More, Right and Better. Jakarta: World Bank; 2016.

[50] Dartanto T Rezki JF Pramono W Siregar CH Bintara U Bintara H Participation of informal sector workers in Indonesia’s national health insurance system. J Southeast Asian Econ. 2016;33:317-42. 10.1355/ae33-3c

[51] Yustina EW Budisarwo J Eddy L The implementation of the national health insurance based on gotong-royong principle as the efforts of enhancing the welfare. Int J Soc Sci Humanit. 2017;7:299-303.

