# Supplementary material for: Impact of Indonesia’s national health insurance scheme on inequality in access to maternal health services: A propensity score matched analysis
Source: J Glob Health. 2020 Jun 11;10(1):010429. doi: 10.7189/jogh.10.010429 (PMC7298736; doi:10.7189/jogh.10.010429)
Supplement: Online Supplementary Document [file jogh-10-010429-s001.pdf]

Online Supplementary Documents

Figure S1. Sample flowchart of 2017 IDHS

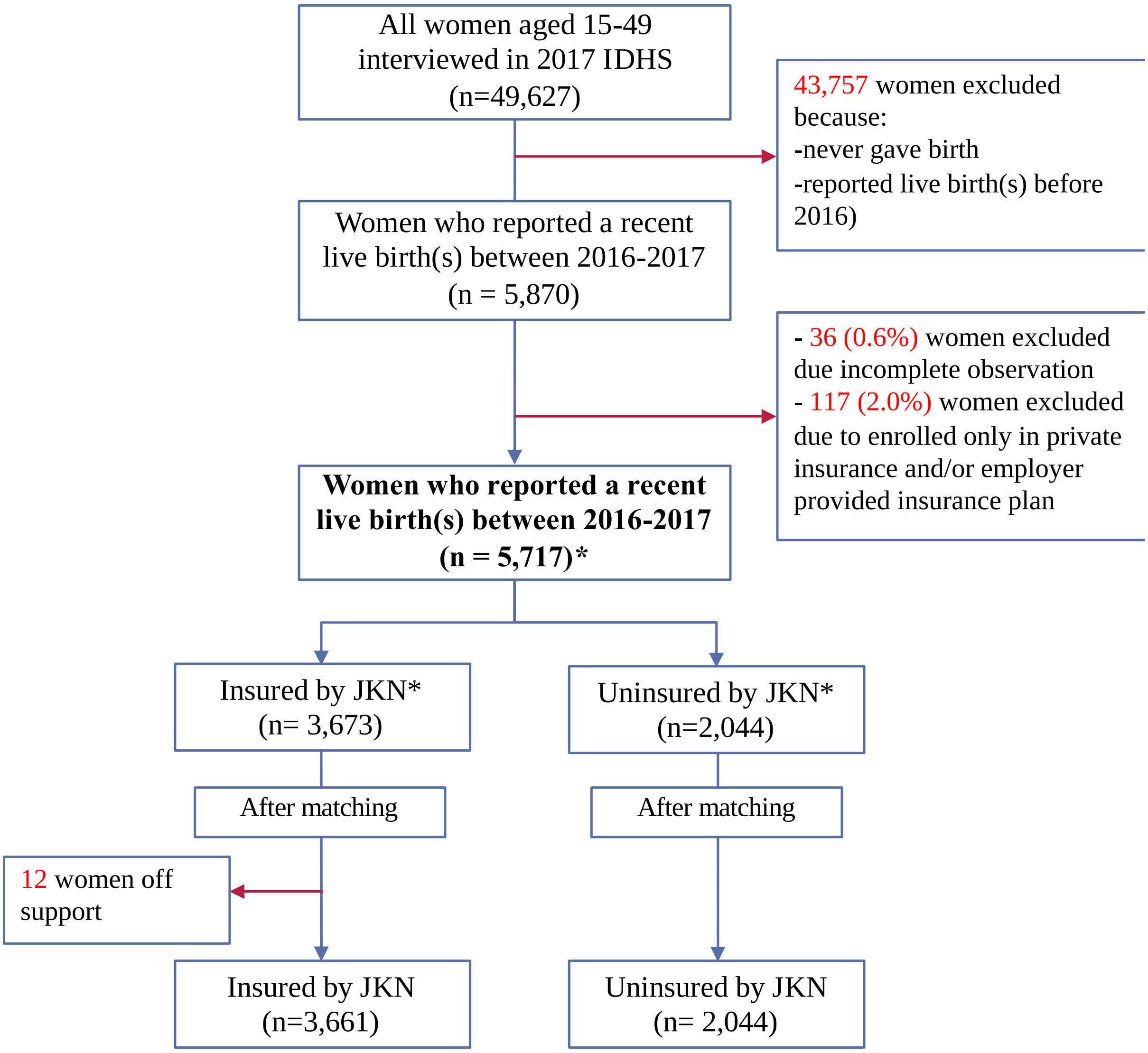

* Insured by JKN: covered by JKN (PBI/Jamkesda (regional health insurance) or non-PBI); Uninsured by JKN: not covered by insurance

Table S1. List of variables

Variables	Type	Measurement	Source of measurement
**Dependent variables**			
a) ANC 4+<br>b) ANC 4+ and received clinical components of ANC[1]<br>c) Skilled birth attendance[2]<br>d) Facility-based delivery[3]<br>e) PNC<br>f) PNC with skilled provider[4]	Binary	0. No<br>1. Yes	ANC 4+ and ANC4+ and received clinical components of ANC<br>• q408: Did you see anyone for antenatal care for this pregnancy?<br>• q409: Whom did you see?<br>• q412B: How many times did you receive antenatal care in: a) the first 3 months?; b) between the fourth and sixth months?; c) between the seventh month and delivery?<br>• Q413: As part of your antenatal care during this pregnancy, were any of the following done at least once?<br>Skilled birth attendance<br>• q429: Who assisted with the delivery of (child's name)?<br>Facility-based delivery<br>• q430: Where did you give birth to (child's name)?<br>PNC with skilled provider<br>• q435: After (child's name) was born, did anyone check on your health while you were still in the facility?<br>• q436: Who checked on your health at that facility?
[1)] Clinical components of ANC include weight and height measured, blood pressure measured, blood and urine sample taken, stomach examined and counselling.<br>[2)] Skilled provider: GP, obstetrician, nurse, midwife, village midwife<br>[3)] Facility-based delivery: hospital, health centre, maternity home, clinic, etc.			
**Main independent variable**			
Enrolment in JKN	Binary	0. Uninsured by JKN (Not covered by insurance)<br>1. Insured by JKN (PBI, Jamkesda and non-PBI)	• q1109: Are you covered by any health insurance?<br>• q1110: What type of health insurance are you covered by?
**Covariates**			
Age (in years)	Categorical ordinal	0. 15-25 years<br>1. 26-35 years<br>2. 36-42 years<br>3. 42-49 years	• q106: How old were you at your last birthday?
Marital status	Binary	0. Unmarried/Divorce<br>1. Married or living together	• q701: Are you currently married or living together with a man as if married?

Variables	Type	Measurement	Source of measurement
			• q702: Have you ever been married or lived together with a man as if married?<br>• q703: What is your marital status now: are you widowed, divorced, or separated?
Birth order	Binary	0. 1<br>1. 2<br>2. 3<br>3. 4<br>4. 5 or more	• q212: What name was given to your (first/next) baby? Record name birth history number
Education	Categorical ordinal	0. None/incomplete primary<br>1. Complete primary<br>2. Incomplete secondary<br>3. Complete secondary<br>4. Higher/vocational	• q107: Have you ever attended school?<br>• q108: What is the highest level of school you attended: primary, junior high, senior high, academy or university?<br>• q109: What is the highest (grade/year) you completed at that level?
Women's occupation	Categorical nominal	0. None<br>1. Agriculture (Agricultural worker)<br>2. Blue-collar (Clerical, sales, industrial worker)<br>3. White-collar (Professional, managers and administration)	• q912: Have you done any work in the last 12 months?<br>• q913: What is your occupation, that is, what kind of work (do/did) you mainly do?<br>• q914: Do you do this work for a member of your family, for someone else, or are you self-employed?
Exposure to internet	Categorical ordinal	0. Not at all<br>1. Less than once in a week<br>2. At least once in a week	q120 Do you use internet in the last 12. Months? q121: In the last months, do you access internet, almost every day, at least once a week, less than once a week or not at all?
Exposure to newspaper	Categorical ordinal	0. Not at all<br>1. Less than once in a week<br>2. At least once in a week	q113: Do you read a newspaper or magazine, at least once a week, less than once a week or not at all?
Wealth index	Categorical ordinal	0. Very poor<br>1. Poor<br>2. Middle<br>3. Rich<br>3. Richest	The wealth index is a composite measure of a household's cumulative living standard. The wealth index is calculated using easy-to-collect data on a household's ownership of selected assets, such as televisions and bicycles; materials used for housing construction; and types of water access and sanitation facilities. The wealth index is presented in the DHS

Variables	Type	Measurement	Source of measurement
			Final Reports and survey datasets as a background characteristic.
Residency	Binary	0. Rural<br>1. Urban	Women-questionnaire:<br>q5: Urban/rural (identified by f interviewers)
Region of residency	Categorical nominal	0. Eastern Indonesia<br>1. Sulawesi<br>2. Kalimantan<br>3. Nusa Tenggara<br>4. Sumatera<br>5. Java & Bali	Women-questionnaire:<br>q1: province (identified by interviewers)

Notes:
Number of questionnaires are based on 2017 IDHS Women-questionnaire

Table S2. Bias reduction in respondents' characteristics using kernel matching

Variables	**Original sample** (before matching, N=5,717) †			**Kernel matched sample** (after matching, N=5,705) †			
	Insured %	Uninsured %	Std diff %	Insured %	Uninsured %	Std diff %	Bias reduction %
Age							
25–34 years	52.8	50.7	4.3	53.0	53.5	-1.1	74.4
35–42 years	22.3	18.1	10.4*	22.2	21.4	1.8	82.2
42–49 years	2.1	1.4	5.3	2.0	2.0	0.7	86.3
Marital status							
Married	98.2	96.8	8.7	98.2	98.2	0	99.5
Birth order							
2	31.2	34.2	-6.4	31.3	30.0	2.8	57.1
3	19.4	19.0	1	19.4	19.2	0.5	52.4
4	10.1	8.9	4.3	10.1	9.8	1.1	75.7
5 or more	7.7	5.4	9.1	7.6	7.6	0	99.6
Education							
Complete primary	15.6	19.1	-9.2	15.7	15.8	-0.5	95
Incomplete secondary	22.7	30.1	-16.8*	22.8	23.0	-0.5	96.8
Complete secondary	31.2	31.8	-1.3	31.3	30.8	1.2	13.5
Higher/vocational	23.4	11.2	32.8*	23.2	23.3	-0.3	99.2
Employment							
Agriculture	8.0	9.2	-4.6	8.0	7.9	0.2	95.2
Blue-collar	23.9	23.4	1.1	23.9	23.8	0.4	67.8
White-collar	17.4	6.4	34.5*	17.1	17.4	-0.9	97.4
Exposure to internet							
Less than once a week	37.7	28.5	19.8*	37.7	38.3	-1.3	93.2
At least once a week	11.0	5.8	18.8*	10.9	10.1	2.8	84.9
Exposure to newspaper							
Less than once a week	14.4	14.3	0.4	14.4	15.3	-2.6	-536.5
At least once a week	80.7	79.6	2.8	80.7	79.7	2.5	10.5
Wealth index							
Poor	19.0	23.2	-10.5*	19.0	19.6	-1.4	86.2
Middle	18.1	18.4	-1	18.1	17.9	0.5	53.5
Rich	18.1	17.6	1.5	18.2	17.8	0.9	41.3
Very rich	18.8	10.6	23.4*	18.6	18.5	0.5	98
Residency							
Urban	51.0	43.3	15.5*	50.9	50.5	0.7	95.5
Region of residency							
Sulawesi	17.9	11.5	18.1*	17.8	17.4	0.9	94.8
Kalimantan	8.1	10.2	-7.4	8.1	7.6	1.4	80.4
Nusa Tenggara	8.2	10.4	-7.5	8.3	8.3	0	99.6
Sumatra	26.8	24.5	5.3	26.9	25.7	2.7	48.2
Java & Bali	28.9	31.9	-6.5	29.0	30.4	-2.9	55.9

Notes:
*Absolute value of mean standardized difference (Std diff) above ±10%.
† Percentages and Ns are **unweighted.**

Table S3. Quality measurements of propensity score matching for enrolment in JKN using kernel matching

	Pseudo-R^2	*P*>chi2	Mean bias	Rubin's B	Rubin's R
Before matching	0.050	<0.0001	9.9	54.7	1.64
After matching	0.001	1.000	1.1	7.4	0.92

Figure S2. Propensity score matching using kernel matching

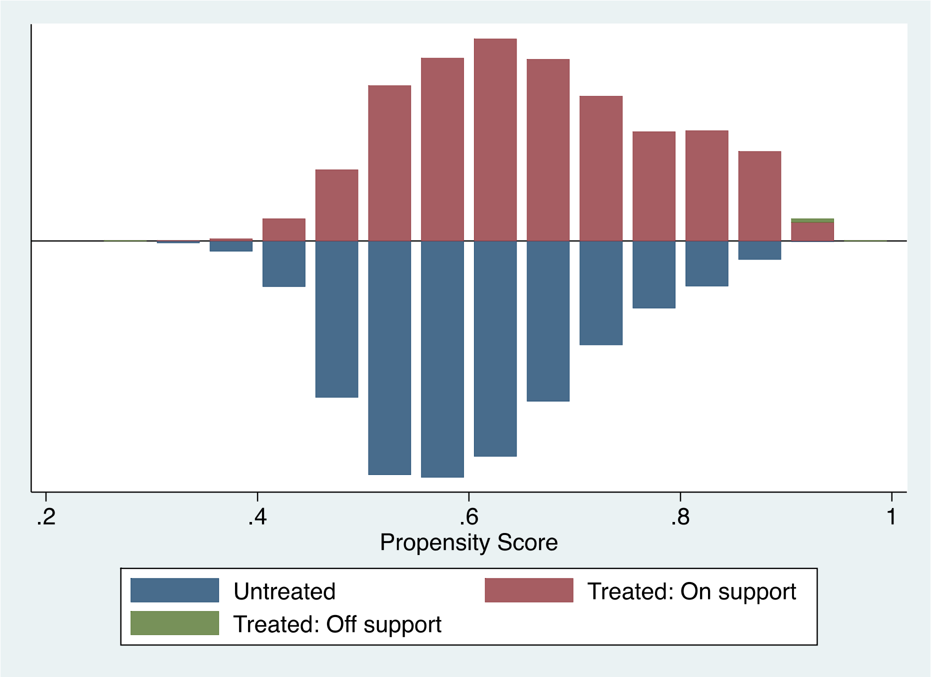

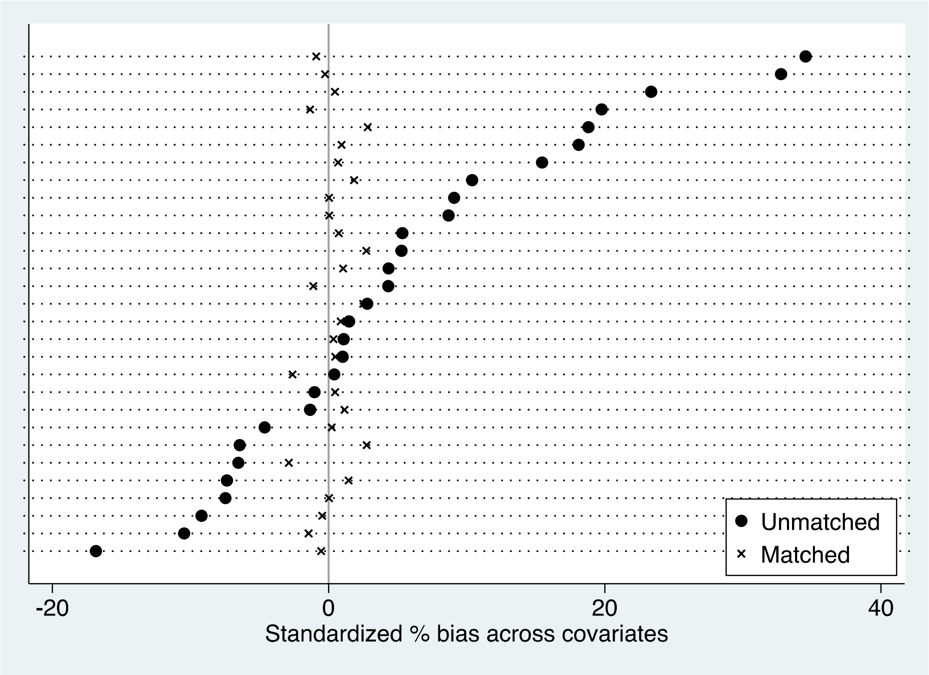

Table S4. Covariate balance across different matching algorithm, according to standardized differences

Variables	Unmatched	NN with Replacement*	NN without replacement*	Radius*	**Kernel**†
	**Standardised differences (5)**				
	N=5,717	N=5,668	N=1,705	N=5,668	**N=5,705**
Age					
25–34 years	4.3	-3	0.3	-1.4	**-1.1**
35–42 years	10.4	2.8	-1.9	2.5	**1.8**
42–49 years	5.3	2.1	-0.4	0.2	**0.7**
Marital status					
Married	8.7	-1.6	-3.6	0	**0**
Birth order					
2	-6.4	4.5	-0.2	2.4	**2.8**
3	1	-0.6	1.8	-0.1	**0.5**
4	4.3	-0.1	0.3	0.4	**1.1**
5 or more	9.1	1.2	-1.2	-0.2	**0**
Education					
Complete primary	-9.2	-1.2	4.8	-0.5	**-0.5**
Incomplete secondary	-16.8	-1.9	3.9	-0.6	**-0.5**
Complete secondary	-1.3	1.1	-3.9	1.3	**1.2**
Higher/vocational	32.8	-0.4	-4.9	-0.3	**-0.3**
Employment					
Agriculture	-4.6	2.9	3.1	0.2	**0.2**
Blue-collar	1.1	3.2	-1.2	0.5	**0.4**
White-collar	34.5	-4.3	-4.9	-0.9	**-0.9**
Exposure to internet					
Less than once a week	19.8	1.3	-4.4	-0.6	**-1.3**
At least once a week	18.8	2.9	-3.5	2.1	**2.8**
Exposure to newspaper					
Less than once a week	0.4	1.4	3.5	-1.1	**-2.6**
At least once a week	2.8	-1.7	-3.6	1.2	**2.5**
Wealth index					
Poor	-10.5	-0.1	3.7	-1.2	**-1.4**
Middle	-1	-0.6	-0.3	0.1	**0.5**
Rich	1.5	0.2	-1.2	1	**0.9**
Very rich	23.4	1.3	-7.6	0.5	**0.5**
Residency					
Urban	15.5	2.1	-4.5	0.7	**0.7**
Region of residency					
Sulawesi	18.1	-0.5	-3.5	-0.1	**0.9**
Kalimantan	-7.4	3.9	3	1.5	**1.4**
Nusa Tenggara	-7.5	4.6	2.6	0	**0**
Sumatra	5.3	1.3	-6.7	3.7	**2.7**
Java & Bali	-6.5	-5.3	1.3	-2.9	**-2.9**

* Calliper width: 0.02

† Bandwidth: 0.2 of the standard deviation of the logit propensity score

Table S5. Background characteristics of women who had recent live birth between 2011 and 2012

Variables	All		Treatment: Insured by JKN		Control: Uninsured by JKN	
	%	N	%	n	%	n
**Overall** *	100	4432	74.5	1129	25.5	3303
**Outcomes variables**						
(a) ANC 4+	69.8	3092	72.9	824	68.7	2268
(b) ANC 4+ and received clinical components of ANC	13.2	585	15.6	176	12.4	409
(c) Skilled birth attendance	85.3	3779	86.3	975	84.9	2803
(d) Facility-based delivery	67.0	2971	68.7	776	66.5	2196
(e) PNC	79.1	3505	80.1	905	78.7	2598
(f) PNC with skilled provider	76.8	3398	77.7	878	76.4	2520
**Control variables**						
Age						
15–24 years	34.0	1508	28.8	325	35.8	1183
25–34 years	48.2	2136	48.7	550	48.0	1586
35–42 years	16.2	717	20.1	227	14.8	490
42–49 years	1.6	71	2.4	27	1.3	44
Marital status						
Unmarried	2.0	90	3.2	36	1.6	54
Married	98.0	4342	96.8	1093	98.4	3249
Birth order						
1	40.3	1786	34.9	394	42.2	1392
2	30.7	1363	31.8	359	30.4	1003
3	15.6	691	16.9	191	15.1	500
4	6.6	294	7.5	84	6.3	210
5 or more	6.7	298	8.9	101	6.0	198
Education						
None/incomplete primary	10.6	471	10.3	117	10.7	354
Complete primary	23.7	1050	25.0	283	23.2	767
Incomplete secondary	29.3	1300	26.4	298	30.3	1002
Complete secondary	25.7	1141	20.5	232	27.5	909
Higher/vocational	10.6	470	17.7	200	8.2	269
Employment						
None	57.2	2534	54.3	614	58.1	1920
Agriculture	9.0	397	7.6	85	9.4	311
Blue-collar	25.4	1125	22.6	255	26.3	870
White-collar	8.5	376	15.5	175	6.1	201
Exposure to newspaper						
Not at all	55.6	2465	52.3	590	56.8	1875
Less than once a week	34.6	1535	33.7	380	35.0	1155
At least once a week	9.7	431	14.1	159	8.3	272
Wealth index						
Very poor	22.5	997	28.1	317	20.6	680
Poor	21.9	968	22.0	248	21.8	721
Middle	20.5	907	19.0	214	21.0	693
Rich	19.9	884	15.9	180	21.3	703
Very rich	15.2	675	15.0	170	15.3	506
Residency						
Rural	55.2	2444	53.5	604	55.7	1840
Urban	44.8	1988	46.5	525	44.3	1462
Region of residency						
Eastern Indonesia	3.6	158	4.6	52	3.2	106
Sulawesi	8.0	356	10.4	118	7.2	238
Kalimantan	5.9	259	6.1	69	5.8	191
Nusa Tenggara	5.3	235	10.6	120	3.5	115
Sumatra	22.3	990	20.0	226	23.1	764

Variables	All		Treatment: Insured by JKN		Control: Uninsured by JKN	
	%	N	%	n	%	n
Java & Bali	54.9	2434	48.2	544	57.2	1890

Notes: This tables includes all women who had birth recent live birth between 2011–2012 before matching.
* Percentages and Ns are weighted. Unweighted sample size = 4,644

Table S6. The average treatment effect on treated (ATT) of *Jaminan Kesehatan Nasional* (JKN) on maternal health services, by using different matching algorithms

Outcomes	ATT (95% CI)				
	Unmatched	NN with Replacement*	NN without replacement†	Radius[1]	**Kernel[2]**
	N=5,717	N=5,668	N=1,705	N=5,668	**N=5,705**
(a) % At least 4 ANC	6.3 (5.0–7.5)***	4.8 (2.7–6.9)***	4.2 (2.7–7.0) ***	5.5 (2.7–8.2) ***	6.0 (3.1–8.7)***
(b) At least 4 ANC and received clinical components of ANC	4.7 (3.6–5.8)***	5.8 (4.0–7.6)***	3.2 (0.8–5.6) ***	5.5 (3.2–7.7) ***	5.6 (3.3–7.9)***
(c) % Skilled birth attendance	4.7 (3.9–5.5)***	3.2 (1.9–4.6) ***	2.8 (0.9–4.7) ***	3.1 (1.4–4.8) ***	3.0 (1.5–4.5)***
(d) % Facility-based delivery	12.2 (11.0–13.3)***	10.8 (8.9–12.8) ***	9.0 (6.3–11.6) ***	10.3 (7.6–12.8) ***	10.2 (7.5–12.7)***
(e) % PNC	5.5 (4.7–6.3)***	4.3 (2.9–5.6) ***	3.9 (2.0–5.8) ***	3.6 (1.9–5.2) ***	3.5 (1.9–5.2)***
(f) % PNC with skilled provider	7.3 (6.3–8.3)***	6.8 (5.1–8.5) ***	5.2 (2.9–7.6) ***	4.7 (2.6–6.7) ***	4.5 (2.6–6.5)***

Significance: *P<0.05; **P<0.01; ***P<0.001
ANC: Antenatal care; PNC: Post-natal care
We applied different matching algorithm as a robustness check: the nearest neighbour with and without replacement, radius matching, and Kernel matching. Kernel matching was selected as the matching algorithm.
* Calliper width: 0.02
† Bandwidth: Bandwidth: 0.2 of the standard deviation of the logit propensity score

Table S7. Background characteristics of sample using PSM and CEM

Variables	Women with recent live birth 2016-2017, original sample (N=5,429)		Women with recent live birth 2016-2017, kernel matched (N=5,425)		Women with recent live birth 2016-2017, coarsened exact matched (N=1,947)	
	Insured	Uninsured	Insured	Uninsured	Insured	Uninsured
**Overall ***	61.4	38.6	61.3	38.7	52.2	47.8
**Outcomes variables**						
(a) ANC 4+	78.6	71.0	78.0	71.0	81.4	77.3
(b) ANC 4+ and received clinical components of ANC	23.4	19.8	23.4	19.8	24.8	21.7
(c) Skilled birth attendance	94.4	90.6	94.4	90.6	94.2	91.9
(d) Facility-based delivery	86.8	78.1	86.8	78.1	88.5	83.4
(e) PNC	90.2	85.4	90.2	85.4	92.4	87.6
(f) PNC with skilled provider	40.3	33.8	37.8	40.4	40.2	36.0
**Control variables**						
Age						
15–24 years	22.9	28.6	22.9	28.6	30.5	30.9
25–34 years	52.4	51.2	52.4	51.2	57.1	58.1
35–42 years	22.4	18.7	22.4	18.7	12.4	11.0
42–49 years	2.3	1.5	2.2	1.5	0.1	0.1
Marital status						
Unmarried	1.6	2.4	1.6	2.4	0.0	0.0
Married	98.4	97.6	98.4	97.6	100.0	100.0
Birth order						
1	32.4	33.2	32.4	33.2	39.3	38.0
2	34.2	37.1	34.2	37.1	40.2	42.5
3	19.5	18.1	19.5	18.1	16.7	15.3
4	8.4	7.5	8.4	7.5	2.1	2.2
5 or more	5.5	4.2	5.5	4.2	1.6	2.0
Education						
None/incomplete primary	6.4	6.7	6.4	6.7	2.1	2.2
Complete primary	16.9	19.9	16.9	19.9	15.4	15.2
Incomplete secondary	25.6	32.6	25.6	32.6	34.2	40.2
Complete secondary	30.9	31.2	30.9	31.2	38.8	34.1
Higher/vocational	20.3	9.6	20.3	9.6	9.5	8.2
Employment						
None	52.7	63.0	52.7	63.0	70.7	74.0
Agriculture	6.2	7.6	6.2	7.6	2.9	2.7
Blue-collar	25.5	24.0	25.5	24.0	19.5	18.0
White-collar	15.6	5.4	15.5	5.4	6.9	5.3
Exposure to internet						
Not at all	57.5	68.4	57.6	68.4	72.0	74.5
Less than once a week	32.7	26.5	32.7	26.5	25.3	23.6
At least once a week	9.8	5.1	9.7	5.1	2.7	1.9
Exposure to newspaper						
Not at all	3.8	4.4	3.8	4.4	1.4	1.4
Less than once a week	13.3	12.3	13.3	12.3	4.4	4.4
At least once a week	82.9	83.3	82.9	83.3	94.2	94.2
Wealth index						
Very poor	19.5	21.4	19.5	21.4	16.2	15.1
Poor	19.2	23.6	19.2	23.6	19.3	23.2
Middle	19.2	20.9	19.2	20.9	24.2	24.9
Rich	20.6	21.1	20.7	21.1	22.4	24.5
Very rich	21.4	13.0	21.4	13.0	17.8	12.3
Residency						

Variables	Women with recent live birth 2016-2017, original sample		Women with recent live birth 2016-2017, kernel matched		Women with recent live birth 2016-2017, coarsened exact matched	
	(N=5,429)		(N=5,425)		(N=1,947)	
	Insured	Uninsured	Insured	Uninsured	Insured	Uninsured
Rural	48.3	57.6	48.4	57.6	47.2	53.0
Urban	51.7	42.4	51.6	42.4	52.8	47.0
Region of residency						
Eastern Indonesia	3.9	2.9	3.9	2.9	2.2	1.7
Sulawesi	8.9	5.0	8.9	5.0	4.3	4.1
Kalimantan	5.2	7.2	5.2	7.2	1.7	2.8
Nusa Tenggara	4.5	5.4	4.5	5.4	2.0	2.2
Sumatra	22.9	22.4	22.9	22.4	19.2	20.1
Java & Bali	54.4	57.0	54.5	57.0	70.6	69.1

Notes:
This tables includes all women who had birth recent live birth between 2016–2017 before matching.
* Percentages and Ns are **weighted**. Unweighted sample size for original sample 5,717 respondents; for kernel matched 5,705; and for coarsened exact matched 1,657.

Table S8. The sample average treatment effect on treated (SATT) of Jaminan Kesehatan Nasional (JKN) on maternal health services using coarsened exact matching

Variables		Treatment group: Insured by JKN	Control group: Uninsured by JKN	SATT*		Relative changes	se
		%	%	%	(95% CI)	%	
(a) % At least 4 ANC visit	Unmatched	74.0	67.7	6.3			1.2
	Matched	76.4	70.9	5.4	(1.5–9.4)***	7%	2.0
(b) % At least 4 ANC and received clinical components of ANC	Unmatched	21.0	16.3	4.7			1.1
	Matched	21.2	17.2	4.0	(0.4–7.7)***	23%	1.9
(c) % Skilled birth attendance	Unmatched	92.7	88.0	4.7			0.8
	Matched	92.5	90.5	1.9	(-0.4–4.3)	2%	1.2
(d) % Facility-based delivery	Unmatched	82.2	70.0	12.2			1.1
	Matched	83.2	76.2	7.0	(3.7–10.3)***	9%	1.7
(e) % PNC	Unmatched	88.4	81.9	6.4			0.9
	Matched	90.2	83.6	6.6	(3.6–9.5)***	8%	1.5
(f) % PNC with skilled providers	Unmatched	38.8	30.1	8.7			1.3
	Matched	38.8	35.0	6.5	(3.1–9.7)***	20%	1.5

Notes:
N after matching = 1,657
ANC: Antenatal care; PNC: Post-natal care; se: standard error
* Significance: ** *P* <0.05; *** *P* <0.01

Table S9. The sample average treatment effect on treated (SATT) of Jaminan Kesehatan Nasional (JKN) on maternal health services, by economic status using coarsened exact matching (CEM)

Subgroups	Treatment	Control	% SATT (95% CI)	*P*-value
At least 4 ANC visits				
Very poor	56.4	52.4	4.0 (-5.2–13.1)	0.402
Poor	76.5	66.5	10.0 (-0.4–20.4)	0.059
Middle	78.3	70.4	7.9 (-0.7–16.4)	0.072
Rich	87.9	83.1	4.8 (-2.4–12.1)	0.191
Richest	91.8	91.4	0.4 (-6.2–7.0)	0.897
At least 4 ANC and received clinical components				
Very poor	5.7	8.4	-2.7 (-7.5–2.0)	0.262
Poor	17.4	18.4	-1 (-9.8–7.9)	0.831
Middle	21.2	21.4	-0.2 (-8.5–8.0)	0.958
Rich	33	21	12.0 (2.9–20.9)	0.010
Richest	35.8	19.2	16.6 (5.4–27.8)	0.004
Skilled birth attendance				
Very poor	76.7	74	2.7 (-4.9–10.1)	0.490
Poor	94.6	90.2	4.4 (-1.7–10.5)	0.157
Middle	98	98.1	-0.1 (-2.9–2.6)	0.928
Rich	98.9	96.1	2.8 (-0.5–6.0)	0.094
Richest	100	100	0 (0–0)	1.000
Facility-based delivery				
Very poor	56.4	48.5	7.9 (-0.9–16.6)	0.075
Poor	85.2	72.9	12.3 (3.3–21.4)	0.008
Middle	90	85.5	4.5 (-0.9–11.6)	0.091
Rich	94.5	88.5	6.0 (0.4–11.7)	0.035
Richest	100	96.6	3.4 (0.4–6.3)	0.025
PNC				
Very poor	80.1	67.0	13.1 (5.4–20.8)	0.000
Poor	96.6	91.2	5.4 (0.08–10.9)	0.153
Middle	98	97.5	0.5 (-2.5–3.5)	0.283
Rich	100	94.2	5.8 (2.4–9.1)	0.035
Richest	97.8	100	-2.2 (-4.9–0.5)	0.735
PNC with skilled provider				
Very poor	71.8	58.8	13.0 (4.5–21.6)	0.003
Poor	91.9	87	4.9 (-2.2–12.1)	0.176
Middle	93.4	91.1	2.3 (-2.9–7.7)	0.381
Rich	95.6	89.3	6.3 (0.9–11.7)	0.022
Richest	97	96.2	0.8 (-3.6–5.1)	0.735

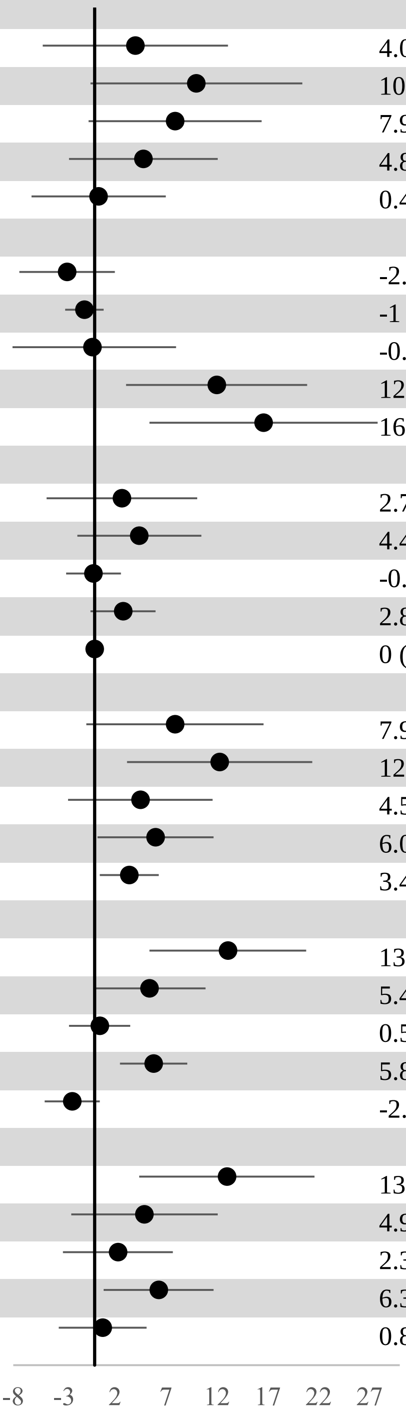

Table S10. The sample average treatment effect on treated (SATT) of Jaminan Kesehatan Nasional (JKN) on maternal health services, by regional of residency using coarsened exact matching (CEM)

**Subgroups**	**Treatment**	**Control**	**SATT (95% CI)**	***P*-value**
At least 4 ANC visits				
Eastern Indonesia	33.3	45.8	-12.5 (-29.4–4.5)	0.149
Sulawesi	71.4	46.8	24.6 (10.9–38.3)	0.001
Kalimantan	86.2	66.7	19.5 (-3.2–42.3)	0.090
Nusa Tenggara	67.4	61.2	6.2 (-17.1–29.5)	0.597
Sumatra	71.2	65.3	5.9 (-2.2–14.2)	0.153
Java & Bali	87.3	84.9	2.4 (-2.3–7)	0.326
At least 4 ANC visits and received clinical components				
Eastern Indonesia	4.8	7.9	-3.1 (-12.5–6.1)	0.499
Sulawesi	11.2	6.6	4.6 (-3.3–12.5)	0.254
Kalimantan	34.5	20.1	14.4 (-11.9–40.6)	0.274
Nusa Tenggara	16.3	17.4	-1.1 (-16.8–14.5)	0.882
Sumatra	10	10.1	-0.1 (-5.6–5.4)	0.972
Java & Bali	32.1	25	7.1 (0.9–13.5)	0.025
Skilled birth attendance				
Eastern Indonesia	66.7	66.7	0 (-16.6–16.6)	1.000
Sulawesi	89.8	80.3	9.5 (0.1–19)	0.048
Kalimantan	100	93.1	6.9 (-3.4–17.2)	0.184
Nusa Tenggara	79.1	74.4	4.7 (-11.5–20.8)	0.567
Sumatra	96.2	93.2	3 (-0.6–6.8)	0.104
Java & Bali	95.7	96.5	-0.8 (-3.4–1.8)	0.533
Facility-based delivery				
Eastern Indonesia	39.7	16.7	23 (8–38.1)	0.003
Sulawesi	69.4	60.3	9.1 (-3.9–22.1)	0.171
Kalimantan	89.7	56.3	33.4 (10.1–56.6)	0.006
Nusa Tenggara	69.8	70.9	-1.1 (-20–17.7)	0.902
Sumatra	83.3	71.7	11.6 (4.4–18.8)	0.002
Java & Bali	94	93.5	0.5 (-2.7–3.7)	0.760
Post-natal care				
Eastern Indonesia	63.5	46.8	16.7 (-0.3–33.7)	0.054
Sulawesi	92.9	78.3	14.6 (4.7–24.5)	0.004
Kalimantan	96.6	58.6	38 (16.8–59.1)	0.001
Nusa Tenggara	76.7	68.6	8.1 (-8.7–25)	0.337
Sumatra	89.2	85.2	4 (-2.1–10)	0.203
Java & Bali	95.2	92.9	2.3 (-0.9–5.5)	0.153
Post-natal care with skilled provider				
Eastern Indonesia	58.7	45.2	13.5 (-3.7–30.7)	0.123
Sulawesi	87.8	74.9	12.9 (2–23.7)	0.020
Kalimantan	96.6	55.2	41.4 (19.9–62.9)	0.000
Nusa Tenggara	74.4	67.4	7 (-10.5–24.5)	0.428
Sumatra	87.9	85.2	2.7 (-3.5–8.9)	0.397
Java & Bali	94.7	91.9	2.8 (-0.5–6.1)	0.098

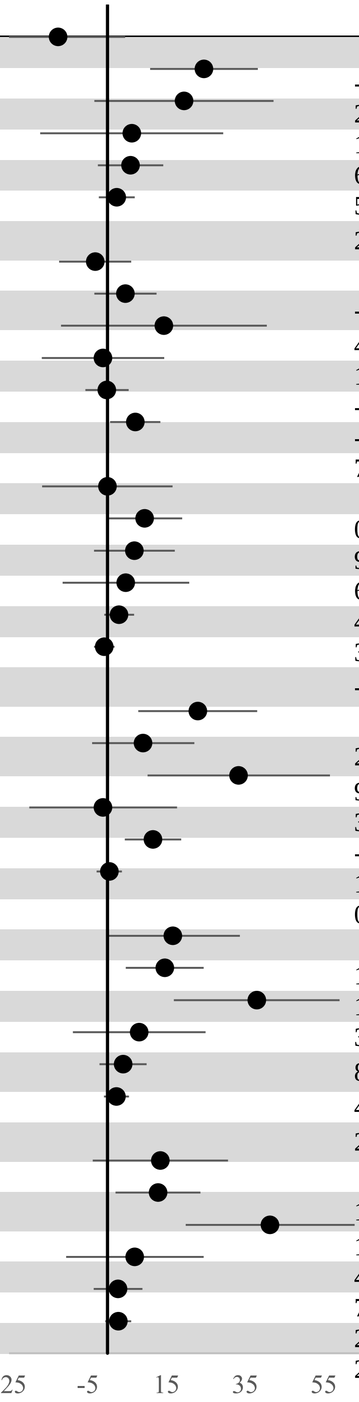

Table S11. Sensitivity analysis using Mantel and Haenzel

**Γ**	**Test statistic**		**Γ**	**Test statistic**	
	**Q_mh+**	***P*_mh+**		**Q_mh+**	***P*_mh+**
	**At least 4 ANC visits**			**Facility-based delivery**	
1.0	4.289	<0.0001	1.0	10.559	<0.0001
1.1	2.965	0.002	1.1	9.052	<0.0001
1.2	1.759	0.039	1.2	7.685	<0.0001
1.3	0.651	0.257	1.3	6.433	<0.0001
1.4	0.301	0.382	1.4	5.279	<0.0001
1.5	1.255	0.105	1.5	4.208	<0.0001
1.6	2.147	0.016	1.6	3.208	<0.0001
1.7	2.988	0.001	1.7	2.270	0.012
1.8	3.781	<0.0001	1.8	1.387	0.082
1.9	4.534	<0.0001	1.9	0.552	0.290
2.0	5.250	<0.0001	2.0	0.175	0.430
	**At least 4 ANC and received essential components of ANC**			**PNC**	
1.0	10.56	<0.0001	1.0	6.267	<0.0001
1.1	9.11	<0.0001	1.1	5.767	<0.0001
1.2	7.78	<0.0001	1.2	4.799	<0.0001
1.3	6.57	<0.0001	1.3	3.916	<0.0001
1.4	5.44	<0.0001	1.4	3.101	<0.0001
1.5	4.40	<0.0001	1.5	2.346	0.009
1.6	3.43	0.0003	1.6	1.642	0.050
1.7	2.52	0.006	1.7	0.981	0.163
1.8	1.66	0.048	1.8	0.359	0.359
1.9	0.85	0.198	1.9	0.137	0.445
2.0	0.79	0.469	2.0	0.695	0.243
	**Skilled birth attendance**			**PNC with skilled providers**	
1.0	4.867	0.000	1.0	7.231	<0.0001
1.1	3.916	0.000	1.1	5.920	<0.0001
1.2	3.047	0.001	1.2	4.730	<0.0001
1.3	2.246	0.012	1.3	3.641	<0.0001
1.4	1.502	0.067	1.4	2.635	0.004
1.5	0.807	0.210	1.5	1.700	0.044
1.6	0.155	0.438	1.6	0.827	0.203
1.7	0.367	0.357	1.7	0.008	0.496
1.8	0.948	0.172	1.8	0.691	0.244
1.9	1.500	0.067	1.9	1.422	0.077
2.0	4.867	<0.0001	2.0	2.117	0.017

Table S12. Multiple hypothesis testing*

Outcome	ATT† (95% CI)	*P*-values Unadjusted	Multiple adj. Bonferroni	Holm
(a) % At least 4 ANC visit	7.4 (4.8–9.9)			
(b) % At least 4 ANC and received clinical components of ANC	5.6 (3.3–7.9)	<0.0001	0.002	0.003
(c) % Skilled birth attendance	3.0 (1.5–4.5)	<0.0001	0.003	0.021
(d) % Facility-based delivery	10.2 (7.5–12.7)	<0.0001	0.002	0.005
(e) % PNC	3.5 (1.9–5.2)	<0.0001	0.002	0.004
(f) % PNC with skilled providers	4.5 (2.6–6.5)	<0.0001	0.002	0.003

* Multiple hypothesis testing was performed using *mulproc* command in Stata

† ATT was estimated using Kernel matching
